# Calvarial Chondroplastic Osteosarcoma With Distant Brain Metastasis Treated With Radiosurgery: A Rare Case Report

**DOI:** 10.1155/carm/5412921

**Published:** 2025-03-18

**Authors:** Mohammed A. Azab, Ahmed Hazim, Nour El-Gohary, Mohsen Nabih Shama, Brahim Kammoun

**Affiliations:** ^1^Department of Neurosurgery, Cairo University Hospital, Cairo, Egypt; ^2^Department of Medicine, Cairo University, Cairo, Egypt; ^3^Department of Emergency Medicine, King Abdullah Medical City, Makkah, Saudi Arabia; ^4^Department of Neurosurgery, Habib Bourguiba University Hospital, Sfax, Tunisia

**Keywords:** calvarium, chemotherapy, metastasis, osteosarcoma

## Abstract

**Background:** Cerebral metastases from soft tissue and bone sarcoma are uncommon. Metastatic sarcoma of the brain is a highly aggressive disease with a poor prognosis. There is no consensus regarding the management of cerebral metastases from bone sarcomas.

**Clinical Presentation:** The patient is a 60-year-old, right-handed male, who presented with a right frontal scalp swelling that was hard in consistency. On examination, he had pain and tenderness over the swelling. The neurological examination was normal.

**Investigations:** Initial CTH revealed a right frontal skull lesion with characteristic expansion and sunburst appearance with a degree of cortical destruction. MRI brain with contrast showed features suggestive of skull osteosarcoma.

**Management:** He underwent a subtotal tumor resection. He was diagnosed with high-grade chondroblastoma-like osteosarcoma of the skull. Subsequently, he received three cycles of neoadjuvant chemotherapy in the form of Adriamycin and cisplatin. One year later, he underwent further surgical intervention with an additional skull resection and reconstruction using mesh and scalp reconstruction.

**Follow-Up:** MRI brain with contrast showed a distant metastasis in the right transverse sinus and other distant brain areas and were treated with Gamma Knife radiosurgery (GKRS) 6 months after the primary surgery.

**Conclusion:** Skull calvarium primary osteosarcoma is a rare pathology. Cerebral metastasis from skull bone osteosarcoma is a challenging clinical situation that requires a multidisciplinary therapeutic approach that includes neurosurgery, plastic surgery, chemotherapy, and radiosurgery.

## 1. Introduction

Primary malignant tumors affecting the bony skull are relatively uncommon compared to skull base chordoma and chondrosarcoma. Osteosarcomas are the most common primary malignant bone tumors, with a high incidence among adults [1]. Osteosarcoma could be associated with Paget's disease, certain genetic syndromes, radiotherapy, or trauma [2, 3]. In 1945, Garland was the first to describe skull osteosarcoma. It is estimated that only 1.6% of all osteosarcomas affect the skull bones [4]. Huvos et al. published a case series of 19 patients with skull osteosarcoma, and only 10 patients had primary de novo tumors [4]. The presentation of skull osteosarcoma is variable and depends upon the location of the tumor. The skull vault is the most affected location [5].

Metastatic bone and soft tissue sarcomas of the brain are highly aggressive diseases with poor prognosis and the survival time ranges from 2 to 7 months [6, 7]. Itoga et al. described only 162 (0.6%) of 26,676 patients with soft tissue sarcoma who had cerebral metastasis [8]. Osteosarcoma accounted for 6% of the cases with sarcomatous brain metastases in the biggest adult series released by the French Sarcoma Group [6]. The true incidence of cerebral metastases from chondroblastic osteosarcoma is not well-defined due to the rarity of these cases [9]. It is well-reported that complete surgical resection with wide surgical margins is associated with a high survival rate [10, 11]. A study in Japan reported a 50% improvement in the Karnofsky Performance Scale (KPS) after the surgical removal of sarcomatoid brain metastasis [12]. For patients treated with gross total resection (GTR), the median overall survival (OS) is variable and ranges from 1.6 to 9.8 months [13, 14]. Chemotherapy, stereotactic radiosurgery (SRS), and whole-brain radiotherapy (WBRT) are other treatment options typically used as adjuvant therapy along with surgery. Moreover, resection of the dura which is commonly involved improves survival and reduces recurrence rates [11]. The standard chemotherapy treatment protocol includes a combination of methotrexate, Adriamycin, and cisplatin [15]. Given the fact that osteosarcoma is radioresistant, radiation therapy is often used for surgically irresectable lesions [5]. We described the rare spread of a local skull bone osteosarcoma to distant brain regions, and we managed to control metastatic disease with radiosurgery, which is reported as a management strategy in only four cases in the literature (Table 1).

## 2. Case Description

**Figure 1:**
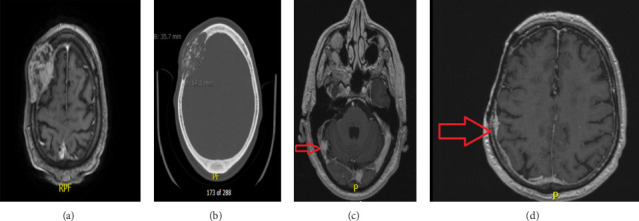
(a, b) Preoperative MRI and CT scan showing the right frontal skull osteosarcoma with characteristic expansion and sunburst appearance with evident cortical destruction. (c) MRI axial images T1 with contrast showing right transverse sinus lesion with heterogeneous intensity and dense contrast uptake. (d) MRI T1 with contrast showing thickened enhanced right-sided dura as evidence for distant metastasis.

**Figure 2:**
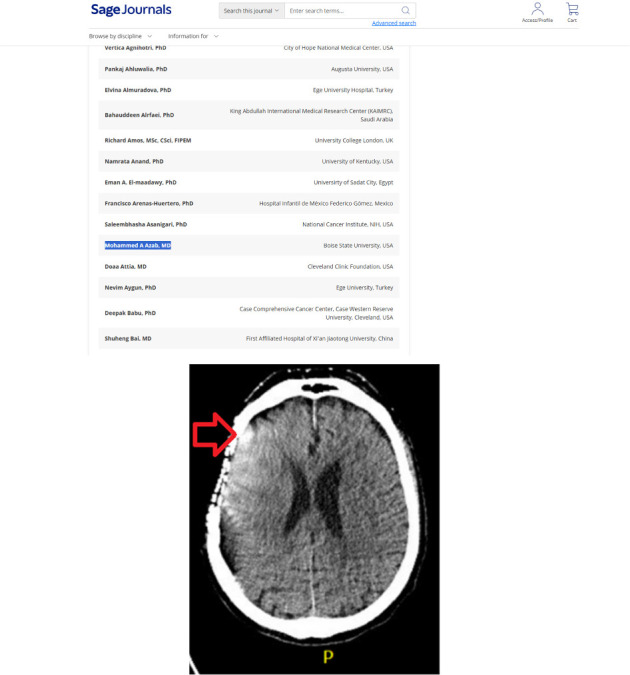
Postoperative CTH showing surgical excision of the calvarium lesion with cranioplasty.

**Figure 3:**
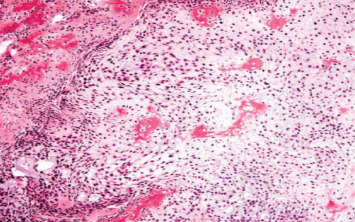
Chondroblastic osteosarcoma. Hematoxylin and eosin stains show malignant chondrocytes with hyperchromatic cytoplasm together with islands of osteoid tissues and spindle cells.

**Figure 4:**
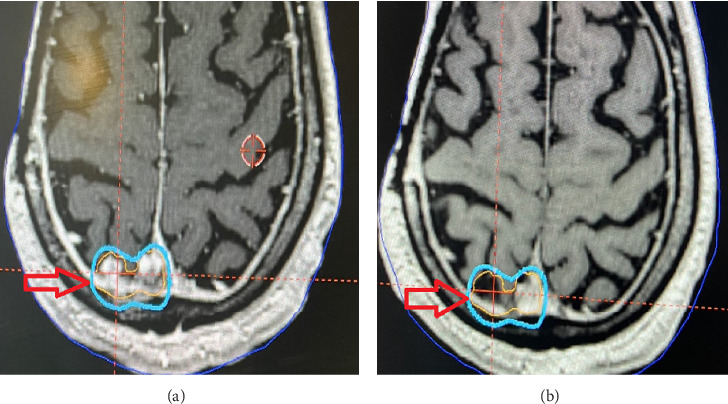
(a) Radiosurgery treatment of one of the cerebral lesions. (b) One year after radiosurgery treatment showed regression of the lesion.

The patient is a 60-year-old, right-handed male, who presented with a right frontal scalp swelling that was hard in consistency. He presented with scalp pain and tenderness over the swelling. Brain imaging was performed that revealed a growing bone lesion with a sunburst appearance (Figure 1). Earlier after diagnosis, he underwent a subtotal tumor resection, and pathology revealed a high-grade chondroblastoma-like osteosarcoma of the right frontal skull. He later received three cycles of chemotherapy with Adriamycin and cisplatin. One year later, further surgical interventions with an additional skull resection and reconstruction using mesh, and scalp reconstruction were done.  Figure 2 shows a postoperative craniectomy defect with cranioplasty mesh. One year later, he presented with a headache associated with a single attack of tonic-clonic seizures. New brain imaging showed a distant metastasis that was observed in the right transverse sinus, right occipital lobe, and the dura. These metastatic lesions were treated with 18 Gy to the 50% isodose line as a single fraction Gamma Knife radiosurgery (GKRS) using a mask.  Figure 3. Six months after SRS, the patient is feeling better with no more headaches. One year follow-up MRI shows regression of the treated lesion (Figure 4).

## 3. Discussion

Cerebral metastases are far more common than primary brain tumors [19, 20]. Cerebral metastases from primary soft tissue and bone sarcoma are uncommon. While the spread of chondroblastoma-like osteosarcoma to the brain is rare, the specific incidence and outcome are not well-defined. About 3% of all brain metastases arise from a primary sarcomatous origin and routine brain surveillance is not systematically required. This concept is changing due to the prolonged survival of those patients and the recent advances in diagnosis and therapy [21]. There are few published case series describing brain metastases from primary bone sarcoma [22–25]. The French Sarcoma Group published a large series of patients with sarcoma brain metastases, and they reported that osteosarcoma-related brain metastases represented only 6% of cases [21]. There are certain histologic subtypes that tend to metastasize to the brain. Malignant fibrous histiocytoma, rhabdomyosarcoma, and leiomyosarcoma are the most frequent pathologies to metastasize to the brain [25]. There is no standardized treatment protocol for the management of brain metastases from sarcoma [22]. Unlike other cerebral metastases, sarcomas are radiochemoresistant tumors [22]. Therefore, surgical resection followed by chemotherapy and radiotherapy is the most accepted treatment for those patients [23].

In this case, primary osteosarcoma affected the right part of the frontal bone with distant spread in different brain locations. Less than 1% of osteosarcomas affect the skull vault, and there are only a few reported cases of skull base osteosarcoma [5]. The typical presentations include bone pain, occasionally accompanied by a mass or by soft tissue swelling [26]. Our patient presented with scalp swelling and pain. The typical radiological features of conventional osteosarcoma are cortical bone destruction, with an overlying periosteal reaction, or a soft tissue mass.

Differentiating de novo skull osteosarcoma from secondary skull osteosarcoma is challenging. However, certain radiopathological features could suggest clues for differentiation. For example, the presence of a soft tissue mass, with outer table thickening and alternating areas of sclerosis and radiolucency, is suggestive of osteosarcoma secondary to Paget's disease [2]. New bone formation with extensive bone destruction is the predominant radiologic criterion of primary osteogenic sarcomas. Secondary osteosarcoma is pathologically like primary osteosarcoma except that it occurs in areas with histological features correlating with the underlying bone disease such as Paget's disease and chronic osteomyelitis. In our case, the images show extensive bone destruction with bone expansion suggestive of primary skull osteosarcoma. The histological examination does not show any features of Paget's disease or radiation-induced changes.

Skull osteosarcoma with cerebral metastasis has an unfavorable response to aggressive multidisciplinary therapy, and the 5-year survival rate is less than 10% [26, 27]. We treated our patient with surgical resection, chemotherapy, and SRS. The total resection of metastases resulted in improved survival compared to patients undergoing subtotal resection [28, 29]. WBRT was used as a traditional treatment for multifocal osteosarcoma brain metastases. However, the unsatisfactory response to usual therapies allowed further evaluation of the role of radiosurgery in this context [30]. Radiosurgery is considered an ablative treatment modality for brain metastases and can be delivered in single or multiple fractions. It could be delivered using different techniques such as Gamma Knife, Cyberknife ,and helical tomotherapy [31]. Several dosing approaches were designed for the treatment of cerebral metastases. According to the American Society for Radiation Oncology (ASTRO) guidelines, a single fraction SRS to a total dose of 20–24 Gy in one fraction is prescribed for patients with metastases < 2 cm in diameter. For patients with lesions measuring 2–4 cm, 27 Gy in 3 fractions or 30 Gy in 5 fractions is recommended, and it is variable between centers depending on the clinical situation and the primary cancer pathology [31, 32].

In this patient, we resected the primary skull lesion and after 6 months, we noticed a distant brain metastasis which we treated with radiosurgery. In a series of radioresistant tumors treated with radiosurgery, nine sarcoma patients with brain metastases were included [33]. The local control rate was 42% and the distant brain failure rate was 56%. The rate of local tumor control was 76% for patients with tumor volume less than 0.5 cm^3^ and 38% for those with tumor volume more than 0.5 cm^3^. The median survival rate was 9 months, and a 1-year survival rate was 22% after SRS. Flannery et al. reported a high local control rate in patients with sarcomatous brain metastatic disease treated with radiosurgery [34]. To the best of our knowledge, this is the first case report of skull calvaria primary chondroblastic osteosarcoma with distant brain metastatic disease treated with radiosurgery. Radiosurgery was effective in treating all the lesions, noticing the gradual regression of the lesions over 12 months of follow-up. In one of the reported cases, recurrence was observed 5 months after surgery close to the resection cavity indicating the need for booster doses to the resection cavity following surgery [16].

## Figures and Tables

**Table 1:** Summary of cerebral lesions secondary to bone osteosarcoma treated with radiosurgery.

Study	No. of patients treated with SRS	Age/sex	Primary location	No. of cerebral lesions	Pathology	Surgical management	SRS treatment	Outcome
Ohla et al. [16]	1	60/F	Left femur	2	Chondroblastic osteosarcoma	Surgical excision of a single lesion	24 Gy was given to the left occipital resection cavity in 3 fractions of 8 Gy each to the 76% isodose line. A single fraction of 20 Gy to the left subinsular lesion was delivered to the 75% isodose line	Intracranial marginal recurrence is superior to the resection cavity of the previously treated occipital lesion at 5 months after surgery
Zamarud et al. [17]	2	NS	NS	NS	NS	NS	18–27 Gy (1–5 fractions)	NS
Murphy et al. [18]	1	9/M	Left femur	1	High-grade conventional osteosarcoma	Surgical resection	The resection cavity was radiated (24 Gy, 3 fractions)	Stable disease was achieved for 7 months before the identification of new intracranial recurrence. A second subtotal resection was performed and then treated with SRS 30 Gy, 5 fractions and palliative oral chemotherapy with etoposide and sirolimus.
Our case	1	60/M	Skull frontal bone	5	High-grade chondroblastoma-like osteosarcoma	Surgical resection of the largest lesion	18 Gy to the 50% isodose line as a single fraction	Stable and controlled intracranial disease

Abbreviations: F, female; M, male; NS, nonspecified; SRS, stereotactic radiosurgery.

## Data Availability

The authors have nothing to report.
